# Impact of Immediate Unilateral Breast Reconstruction with Abdominal Flaps on Quality of Life: A Single-Center Prospective Interventional Study in Egypt

**DOI:** 10.1007/s00266-025-04843-7

**Published:** 2025-04-28

**Authors:** Sherif G. Amr, Ashraf A. Khater, Mohammed H. El Fahar, Ahmad A. Khalil, Ahmed H. El Sabbagh

**Affiliations:** 1 Specialist of Plastic Surgery at Mansoura International Hospital, Mansoura, Egypt; 2 Faculty of Medicine, Surgery, Oncology Centre, Mansoura University, Mansoura, Egypt; 3 Plastic and Reconstructive Surgery, Faculty of Medicine, Mansoura University, 60 El Gomhoria St, Mansoura, 35516 DK Egypt

**Keywords:** Breast cancer, Breast reconstruction, DIEP, Abdominal flap, Quality of life, Delay concept

## Abstract

**Introduction:**

Breast cancer is a significant global health challenge, particularly in Egypt, where mastectomy rates have risen due to increased awareness and advancements in breast imaging. This study investigated the impact of immediate breast reconstruction following mastectomy on quality of life (QoL) in Egyptian women during the COVID-19 pandemic. The research examined three abdominal flap techniques and their effect on patient QoL. Recognizing the scarcity of prior research in Egypt on advanced breast reconstruction and the limited implementation of multidisciplinary care models, this study aimed to address this gap in the literature.

**Patients and Methods:**

This single-center, prospective, non-randomized interventional study, conducted in Egypt between August 2020 and August 2022, involved 36 female patients who underwent breast reconstruction following mastectomy. Participants were classified into three groups (pedicled MS-TRAM flap, free DIEP flap, and delayed free DIEP flap) based on flap selection, guided by surgeon and patient counseling. While surgical techniques varied, preoperative assessments and postoperative care were standardized across all groups. QoL was assessed using the BREAST-Q assessment tool, with one assessment occurring 12 months after surgery.

**Results:**

This study showed that the three surgical groups demonstrated similar QoL outcomes, as assessed by the BREAST-Q. There were no significant differences in satisfaction with breast appearance, psychological well-being, sexual well-being, physical well-being of the abdomen, or physical well-being of the chest and upper body (*p* > 0.05 for all comparisons). Operative time was significantly longer for the free DIEP flap group compared to the MS-TRAM and delayed free DIEP groups (P = 0.001). Postoperative complications were infrequent and comparable across the groups, with no significant differences in total flap necrosis, partial flap loss, or fat necrosis (P =1.0 for all comparisons). The use of mesh support varied significantly among the groups, with 100% utilization in the MS-TRAM group compared to 34% in the free DIEP group and 16.7% in the delayed free DIEP group (P = 0.015 and P = 0.06, respectively).

**Conclusion:**

In this Egyptian study, immediate unilateral ABR using different abdominal flap techniques resulted in similar QoL outcomes using BREAST-Q. This suggests that flap choice does not significantly impact patient well-being and satisfaction. Despite the limitations of this study, it emphasizes the importance of individualized surgical approaches based on patient needs and surgeon experience, particularly in resource-constrained settings. Further research is needed to validate these findings and explore long-term outcomes.

**Level of Evidence II:**

This journal requires that authors assign a level of evidence to each article. For a full description of these Evidence-Based Medicine ratings, please refer to the Table of Contents or the online Instructions to Authors at www.springer.com/00266.

## Introduction

A woman’s breasts are an essential part of her body image, and a breast cancer diagnosis can significantly impact that image, potentially leading to lifelong struggles [1, 2]. Breast cancer constitutes a significant global health challenge, with 2,296,840 new cases reported in 2024. This accounts for 11.5% of all new cancer diagnoses worldwide, making it the most prevalent cancer among females and the second most common cancer overall, following lung cancer [3]. The situation in Egypt is particularly alarming, with a high incidence rate of breast cancer. Data from the National Cancer Registry of Egypt show an incidence rate of 55.4 per 100,000 women, accounting for 34.9% of all new female cancer cases. This highlights the disproportionate burden of breast cancer in Egypt and underscores the urgent need for effective prevention and treatment strategies [3, 4]. Trends in mastectomy rates vary based on factors such as population and stage of breast cancer. A study by Kummerow et al. found that mastectomy rates for early-stage breast cancer in the USA declined until 2005 but have since increased. This shift is likely due to a combination of increased awareness, advancements in breast imaging, and improved detection and screening rates [5]. Following a mastectomy, whether necessitated by breast cancer or prophylactic due to gene mutation, patients may elect to undergo breast reconstruction. The objective of this procedure is to restore the external form of the breast and thereby enhance the patient’s overall quality of life [6]. Both implant-based and autologous breast reconstruction (ABR) are options for restoring breast form after mastectomy [7]. This study focuses specifically on ABR techniques. This approach offers potential advantages in terms of natural appearance and long-term outcomes and reduced risk of complications of implant-based reconstruction [8, 9]. Multiple studies have investigated the relative merits of each reconstructive method in terms of patient satisfaction and overall QoL. These studies suggest that ABR might be accompanied by higher degrees of satisfaction with the reconstructed breast, improved sexual and psychosocial well-being, and a more favorable overall outcome compared to implant-based reconstruction [10, 11]. The ideal breast reconstruction method has to be safe, valid, accessible to all patients, and cause minimal damage to the donor site [12]. Hartrampf pioneered the transverse rectus abdominis myocutaneous (TRAM) flap in 1982, establishing it as a promising option for ABR reconstruction [13]. The deep inferior epigastric perforator (DIEP) flap, a modified TRAM flap created by Allen and his colleague, is now a popular technique for breast reconstruction using the patient’s tissue [14]. Both techniques transfer abdominal skin and fatty tissue for ABR, with special attention to preserving the underlying abdominal muscles. While DIEP and muscle-sparing TRAM (MS-TRAM) flaps share comparable aesthetic results, their technical aspects and potential effects on the abdominal wall diverge [15]. The development of bi-pedicled or stacked abdominal flaps addresses the problem of inadequate donor tissue for abdominal-based perforator flap breast reconstruction in low BMI patients undergoing unilateral procedures [16, 17]. Bilateral breast reconstruction often necessitates more complex approaches. These include bilateral stacked flaps, and four-flap reconstructions incorporating free flaps from separate donor sites [18, 19]. Beugels et al. used a delay procedure to augment the DIEP flap territory by recruiting additional tissue from the flank presenting a compelling alternative for tissue reconstruction [20]. Assessing QoL after breast reconstruction is crucial to determine the overall success of the procedure. Tools like the BREAST-Q help gather patient-reported outcome measures (PROMs), providing valuable data on QoL, encompassing physical and mental well-being and breast satisfaction [21]. This study investigated the impact of immediate breast reconstruction following mastectomy on quality of life (QoL) in Egyptian women during the COVID-19 pandemic. The research examined three abdominal flap techniques and their effect on patient QoL, as measured by the BREAST-Q. Recognizing the scarcity of prior research in Egypt on advanced breast reconstruction and the limited implementation of multidisciplinary care models, this study aimed to address this gap in the literature.

## Patients and Methods

This single-center, prospective, non-randomized cohort study was conducted at a tertiary university hospital in northern Egypt between 2020 and 2022 and evaluated various surgical approaches for unilateral immediate autologous breast reconstruction (ABR) in 36 female patients aged 18 to 60 years with a body mass index (BMI) of 28 to 35 kg/m^2^. Patients were stratified into three groups (n = 12 per group): Group A underwent reconstruction using a pedicled muscle-sparing transverse rectus abdominis myocutaneous (MS-TRAM) flap, Group B with a free deep inferior epigastric perforator (DIEP) flap, and Group C with a delayed free DIEP flap. Exclusion criteria included: age > 60 years, comorbidities (e.g., diabetes mellitus, cardiac disease, hepatic insufficiency, renal dysfunction), morbid obesity (BMI ≥ 40), prior abdominal surgery with median or paramedian incisions, active tobacco use within 3 months preoperatively, bilateral or delayed breast reconstruction indication, history of radiation therapy, and unrealistic expectations of surgical outcomes.

### Sample Size

The sample size calculation was based on the incidence of immediate unilateral breast reconstruction with abdominal flaps. Using G*Power program version 3.1.9.7, with an effect size of 0.86, a two-tailed test, an alpha error of 0.05, and a power of 80%, the minimum calculated sample size supposed to be 10 in each group, supporting the adequacy of our sample size to detect clinically meaningful differences between the study groups. A post hoc power analysis confirmed that our study achieved a power of 80% to detect clinically meaningful differences between the groups.

### The Choice of the Abdominal Flap for Reconstruction

It was determined through a shared decision-making process between the surgeon and the patient. This process involved a thorough discussion of the available surgical options, their potential benefits and risks, and the patient’s individual preferences and expectations. The surgeon’s expertise and comfort level with different techniques, as well as the availability of resources, were also considered in the final decision.

### Preoperative Assessment

Preoperative assessment included the patient’s age, BMI, smoking condition, and the existence of comorbidities. The selection of the reconstructive technique was determined by surgeon preference, patient preference following thorough counseling, and the availability of resources, which was occasionally influenced by the constraints imposed by the COVID-19 pandemic. Preoperative assessment of the donor site included photographic documentation, computed tomography angiography (CTA), and Doppler ultrasound (US) with a handheld Doppler probe (10 MHz). Laboratory investigations included a full blood count, coagulation profile, blood sugar level, and liver and kidney function tests.

### Surgical Technique

All procedures were performed under general anesthesia with the patient-positioned supine and arms abducted at 90 degrees. They were conducted by two specialized surgical teams: one focusing on oncological surgery (oncosurgeon team) and the other on plastic and reconstructive surgery (plastic surgeon team). The oncosurgeon team performed the mastectomy and any necessary lymph node dissection, while the plastic surgeon team performed the DIEP flap reconstruction. Three different surgical approaches were used for immediate autologous breast reconstruction (ABR):

#### Group A: Pedicled MS-TRAM Flap

The contralateral pedicled MS-TRAM flap was harvested according to the technique described by Alisha Fong et al. [22]. The flap was then shaped to create the breast mound, with appropriate areas deepithelialized to optimize the contour. The rectus sheath was closed with a prolene mesh, and the donor site was closed primarily.

#### Group B: DIEP Flap

A DIEP flap was harvested as described by Allen and Treece [14]. Briefly, an 8–12-cm pedicle, encompassing the deep inferior epigastric artery (DIEA) and venae comitantes, was meticulously dissected. The flap was transferred to the chest wall, and microvascular anastomoses were performed, connecting the DIEA to either the internal mammary or thoracodorsal artery. The accompanying venae comitantes were anastomosed to suitable recipient veins. The superficial inferior epigastric vein (SIEV) was also dissected and preserved for potential use in augmenting venous drainage. Following successful microvascular anastomoses, the flap was inset and contoured to create the breast mound. Abdominal wall closure was achieved with primary closure of the rectus sheath and plication of the anterior rectus sheath. The abdominal donor site was closed in conjunction with a full abdominoplasty. Two surgical drains were placed at the breast incision site before closure.

#### Group C: Delayed DIEP Flap

This group underwent a two-stage delayed DIEP flap reconstruction.

**Fig. 1:**
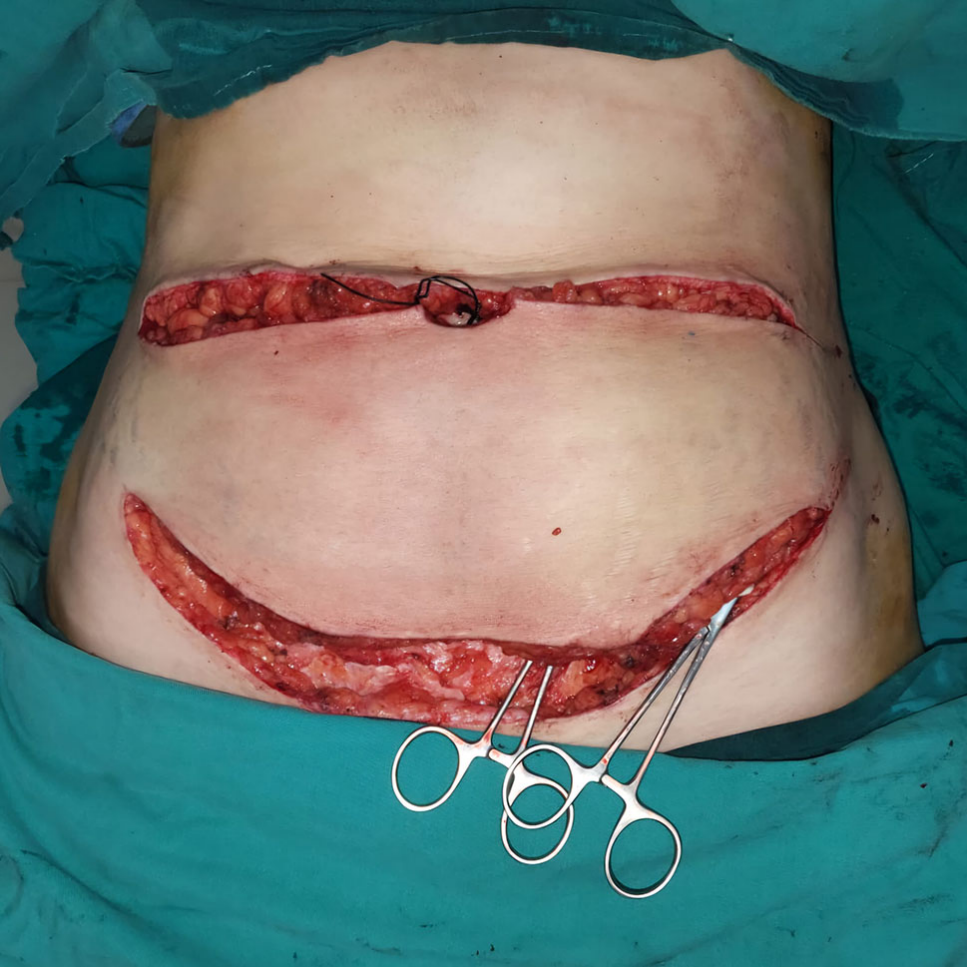
(Stage 1) The lower abdominal flap was incised as traditional DIEP flap as part of the first stage of delayed flap design with 4-cm bridge preservation on both sides

**Fig. 2:**
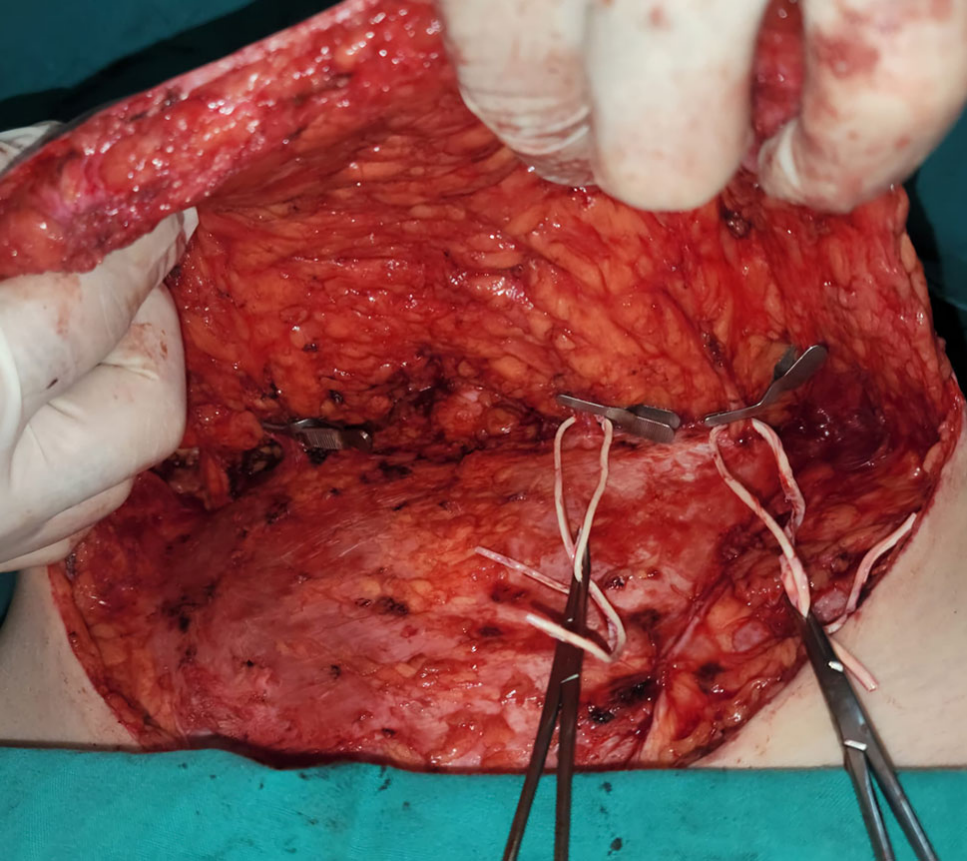
(Stage 1) Intraoperative photograph demonstrating the dissection of the dominant perforators of the DIEP flap; the minor perforators are ligated

**Fig. 3:**
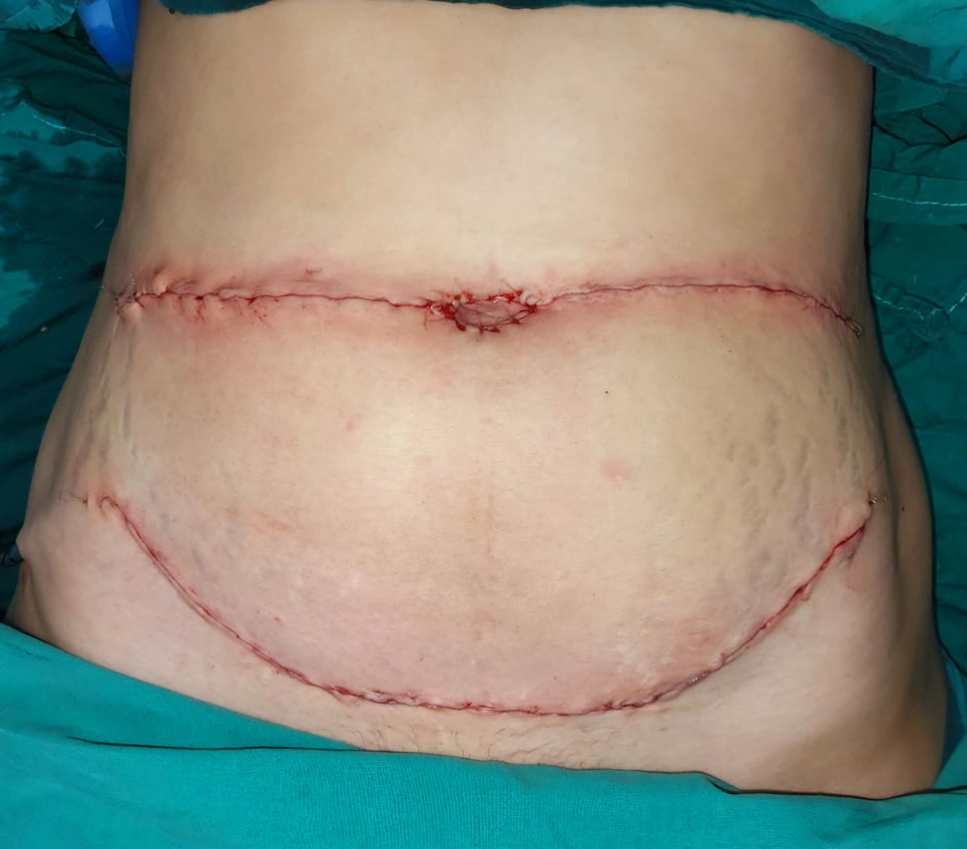
**(**Stage 1) Skin closure over the dissected DIEP flap with two drains on both sides

*Stage 1 (Delay):* Preoperatively, the dominant DIEP perforator was identified through meticulous mapping across a classic DIEP flap. This flap extended to the anterior superior iliac spine. A 4-cm bridge was preserved on both sides of the abdomen (Fig. 1), similar to the design described by Beugels et al. [20] The procedure was performed without ICG angiography or magnetic resonance angiography (MRA). The abdominal flap was then meticulously dissected superficial to the anterior rectus sheath, preserving the selected dominant perforator(s) for vascular supply. All minor perforators on both sides were ligated and divided (Fig. 2). Hemostasis was achieved, and the skin was closed over the dissected flap (Fig. 3).

**Fig. 4:**
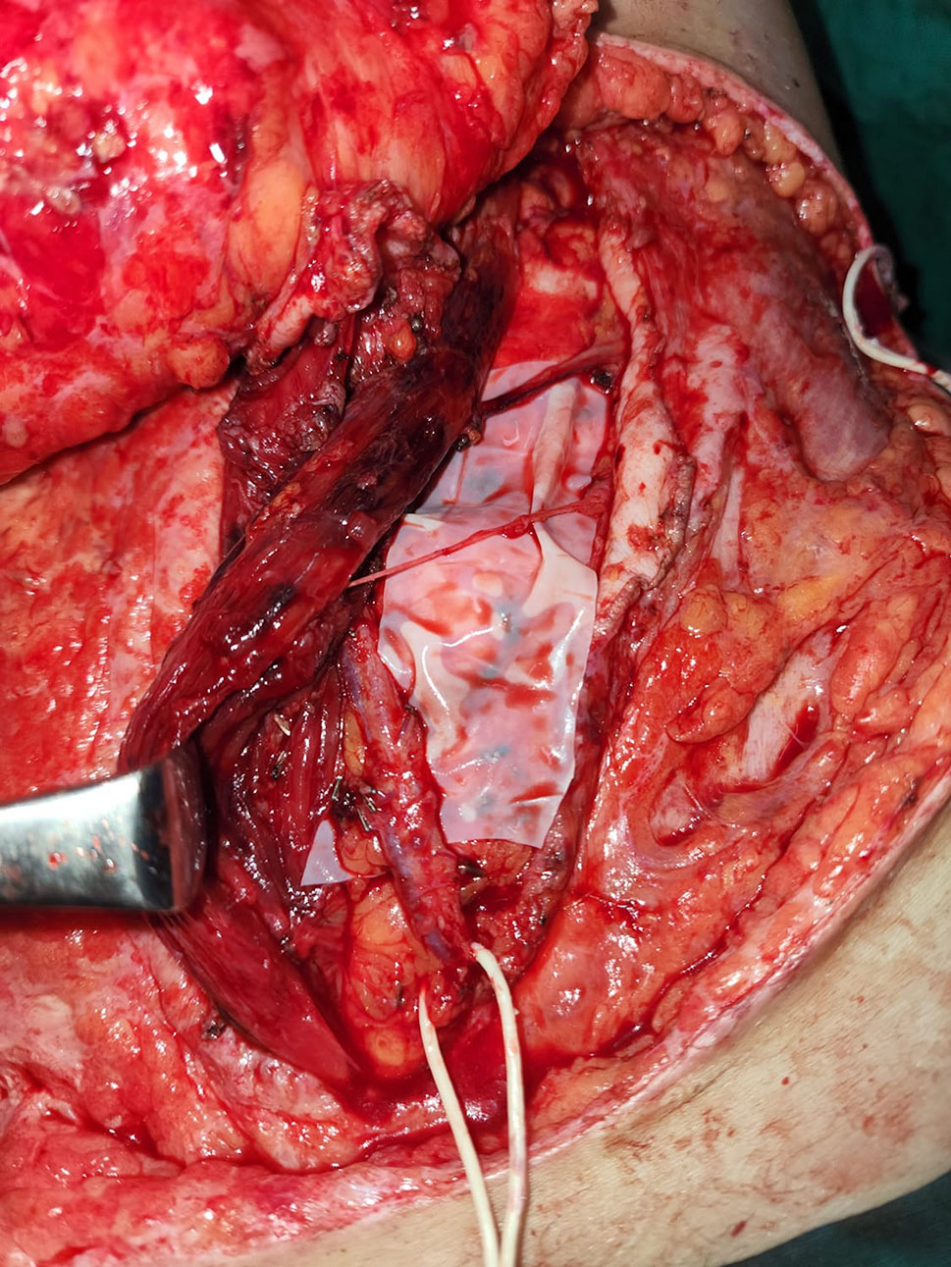
(Stage 2) Two weeks after the initial procedure, the delayed DIEP flap was raised. The DIEP vessels were dissected, fully separating the flap and preparing it for transfer

*Stage 2 (Reconstruction performed 2 weeks later):* The previously dissected flap was elevated, and the perforators were meticulously dissected to ensure adequate exposure while preserving the nerve supply to the rectus muscle (Fig. 4). The remaining steps were carried out as per the typical DIEP flap insertion and abdominal wall closure protocol.

### Postoperative Care

Following surgery, a loosely applied dressing was placed over the breast incisions. Patients were positioned supine with the head of the bed elevated at thirty degrees to minimize tension on the abdominal closure. Four drains were strategically placed in each patient: two in the abdominal donor site and two in the reconstructed breast (one superior and one inferior to the flap). Postoperative drain management was guided by drainage volume, with removal typically occurring between 7 and 10 days at the surgeon’s discretion. Generally, drains were removed when output was consistently below 30–40 mL per 24 h for two consecutive days. Drain output was closely monitored, and removal was performed by the attending surgeon. Early ambulation was encouraged with slight flexion at the waist for the first few days postoperatively to facilitate wound healing. For the first 48 h, flap monitoring was done every two hours; for the next 48 h, it was done every four hours; and for the remaining two weeks, it was done every six hours. Monitoring modalities included handheld Doppler assessment of vascularity, flap temperature, capillary refill time, and flap color.

### Postoperative Analgesia and Medication

In the immediate postoperative period, pain was controlled with intravenous opioid analgesics for the first 24 hours. Patients were then transitioned to a combination of oral ibuprofen 400mg twice daily and intravenous acetaminophen 1000 mg twice daily for ongoing analgesia. A standard antibiotic regimen of intravenous ampicillin–sulbactam, gentamicin, and metronidazole was administered for five days. Intravenous fluid administration was titrated based on central venous pressure monitoring. For thromboprophylaxis, patients received subcutaneous low molecular weight heparin (0.5–1 mg/kg/24 h).

### Postoperative Assessment

This study evaluated patients for 12 months following surgery, with comprehensive postoperative assessments. Data collected included patient demographics, operative reports, and any postsurgical sequelae, including adverse events at the recipient and donor sites and other complications. Patient-reported outcomes (PROs) were assessed at 12 months using the BREAST-Q, a validated instrument for measuring patient experience and satisfaction after breast surgery. BREAST-Q data, initially scored on a 1–5 scale, were converted to a 0–100 scale using Q-Score software, with higher scores indicating greater patient satisfaction and quality of life (QoL) [21].

### Statistical Analysis

SPSS software, version 25, was used for statistical analysis. Frequencies and percentages were used to present qualitative data. Mean ± SD was used for quantitative data with a normal distribution, while median (IQR) was used for data that were not normally distributed. The Chi-square test was used to compare qualitative data, and the Student’s t test was used for normally distributed quantitative data. One-way ANOVA followed by a post hoc test was used to compare normally distributed quantitative data across multiple groups. Non-normally distributed quantitative data were compared using the Mann–Whitney U test or the Kruskal–Wallis test. Spearman’s test was used to analyze the correlation between non-normally distributed quantitative data. A significance level of *p *< 0.05 was used.

## Results

This study included 36 participants with a mean age of 38.6 ± 6.09 SD years. Most participants (72.2%) had a history of lower abdominal surgery. Participants were divided into three groups of 12. The groups were similar in terms of age, with Group A averaging 41.83 ± 2.32 years, Group B 37.17 ± 5.38 SD years, and Group C 38.0 ± 6.32 SD years. There were no significant differences in BMI between the groups (p = 0.518). The average BMI was 32.45 kg/m^2^ for Group A, 30.58 kg/m^2^ for Group B, and 32.57 kg/m^2^ for Group C. The proportion of participants with a history of lower abdominal surgery was similar in Groups A and B (34% each) compared to Group C (16.7%). Breast size distribution was also comparable across the groups: 50% B cup in Groups A and B and 34% in Group C. This observation indicates that breast size was not a confounding factor in the analysis of outcomes.

Half of the participants in Group A had right breast involvement, compared to two-thirds in Groups B and C. Overall, the three groups were highly similar in terms of age, BMI, history of lower abdominal surgery, breast size, and laterality of breast involvement. No statistically significant differences were observed between the groups for any of these factors (*p *> 0.05 for all comparisons)**.**

Our cohort reveals a predominance of invasive mammary carcinoma (58.30%) among the diagnosed breast cancer types, followed by invasive lobular carcinoma (27.80%) and DCIS (13.90%). Surgically, modified radical mastectomy was the most frequent procedure (52.80%), closely followed by axillary clearance (58.30%), reflecting the importance of assessing and addressing lymph node involvement. Sentinel lymph node biopsy was performed in 41.70% of cases, suggesting a trend toward more targeted and less invasive staging. While mastectomy combined with SLN biopsy also represented 41.70% of cases, the lower number of skin-sparing mastectomies (5.60%) indicates this approach was less common. In terms of treatment strategy, upfront surgery was favored in 63.90% of cases, while neoadjuvant therapy was utilized in 36.10% of cases. These findings highlight the distribution of breast cancer subtypes and the surgical and treatment approaches employed within the studied population. In our study, 13 cases received 8 sessions of neoadjuvant therapy and underwent surgery within 2 weeks of completing the therapy. This timeframe was chosen to minimize the delay between completing neoadjuvant therapy and surgical intervention while allowing sufficient time for patients to recover from the effects of the therapy **(**Table 1**).**

**Table 1:** Patients’ demographics between the three groups

Characteristic	Category	Group A (N=12)	Group B (N=12)	Group C (N=12)	Total (N=36)	%	Test of significance	Within-group significance
Age (Years)	Mean ± SD	41.83 ± 2.32	37.17 ± 5.38	38.0 ± 6.32			F = 1.5, P = 0.255	P1 = 0.150, P2 = 0.08, P3 = 0.709
BMI (kg/m^2)	Mean ± SD	32.45 ± 3.14	30.58 ± 3.61	32.57 ± 3.08			F = 0.688, P = 0.518	P1 = 0.125, P2 = 0.202, P3 = 0.776
Lower abdominal operation	Negative	4 (34%)	4 (34%)	2 (16.7%)	10	27.80%	χ^2^ = 0.23, P = 1.0	P1 = 1.0, P2 = 1.0, P3 = 1.0
	Positive	8 (66%)	8 (66%)	10 (83.3%)	26	72.20%		
Breast size	B	6 (50%)	6 (50%)	4 (34%)	16	44.40%	χ^2^ = 0.34, P = 1.0	P1 = 1.0, P2 = 1.0, P3 = 1.0
	C	6 (50%)	6 (50%)	8 (66%)	20	55.60%		
Breast side	Right	6 (50%)	8 (66%)	8 (66%)	22	61.10%	χ^2^ = 0.355, P = 1.0	P1 = 1.0, P2 = 1.0, P3 = 1.0
	Left	6 (50%)	4 (34%)	4 (34%)	14	38.90%		
*Surgical procedures*								
Invasive mammary carcinoma		6	8	7	21	58.30%		
Invasive lobular carcinoma		4	3	3	10	27.80%		
Ductal carcinoma in situ (DCIS)		2	1	2	5	13.90%		
Modified radical mastectomy		8	6	5	19	52.80%		
Skin-sparing mastectomy		0	1	1	2	5.60%		
Mastectomy + SLN biopsy		4	5	6	15	41.70%		
Axillary clearance		8	7	6	21	58.30%		
Sentinel lymph node biopsy		4	5	6	15	41.70%		
Neoadjuvant therapy		5	4	4	13	36.10%		
Upfront surgery		7	8	8	23	63.90%		

There was a significant difference in operative time between the three groups (F = 17.72, P = 0.001). Post hoc analysis showed that Group B had a significantly longer operative time (6.0 ± 0.71 hours) than Group A (9.08 ± 1.42 h) and Group C (6.33 ± 0.61 h) (P = 0.001). There was no significant difference in operative time between Group A and Group C (P = 0.566). There was no significant difference in the length of hospital stay between the three groups (F = 2.02, P = 0.167). Group B had an average ischemic time of 42.50 ± 14.04 min, while Group C had an average ischemic time of 31.33 ± 7.78 min. The difference between the two groups was not statistically significant (t = 2.901, P = 0.119). Group B had an average perforator diameter of 2.72 ± 0.47 mm, while Group C had an average perforator diameter of 2.88 ± 0.53 mm. The difference between the two groups was not statistically significant (t = 0.334, P = 0.576). There was no significant difference between Group B and Group C in the number of patients who underwent anastomosis with internal mammary vessels or thoracodorsal vessels (χ2 = 0.444, P = 0.505) **(**Table 2**).** Postoperative complications were infrequent and comparable across the three groups. Total flap necrosis occurred in 2 patients (16.7%) from Group A and 2 patients (16.7%) from Group B (p = 1.0). For patients who experienced total flap necrosis, the authors performed a pedicled latissimus dorsi myocutaneous flap to salvage the reconstruction and achieve the best possible outcome. Patients with failure were included in the QoL analysis, as their experiences and outcomes are still valuable in understanding the overall impact of breast reconstruction. Similarly, partial flap loss affected 2 patients (16.7%) in both Group A and Group B (p = 1.0). These cases were managed with either debridement and primary closure or dressings with secondary intention. Fat necrosis was observed in 2 patients (16.7%) from each of Group A and Group B (p = 1.0) and was addressed with debridement and either vacuum-assisted closure (VAC) therapy or primary closure. Venous congestion occurred in 4 patients (34%) in Group A and 4 patients (34%) in Group B (p = 1.0). Abdominal comorbidities were observed in two patients (16.7% of Group A), both of whom had incisional hernias. These hernias were managed conservatively using supportive binders and analgesics. Surgical intervention was offered to both patients; however, both declined. Four patients (34%) in Group B required reoperation for venous congestion. All cases were treated with SIEV supercharging. Two patients experienced hematoma formation, which directly compromised the anastomosis, necessitating revision. In the other two patients, venous congestion developed rapidly (within 24 h), impeding graft outflow and requiring re-exploration and revision with SIEV supercharging to re-establish patency.

**Table 2:** Operative details

	Group A N=12	Group B N=12	Group C N=12	Test of significance	Within-group significance
Length of hospital stay/days mean ± SD	8.0±1.41	9.33±1.75	9.67±1.37	F=2.02 P=0.167	P1=0.150 P2=0.08 P3=0.709
Operative time in hours mean ± SD	6.0±0.71	9.08±1.42	6.33±0.61	F=17.72 P=0.001*	P1=0.001* P2=0.566 P3=0.001*
Ischemic time/minutes mean ± SD	0	42.50±14.04	31.33±7.78	t=2.901 P3=0.119	
Diameter of perforator/mm	0	2.72±0.47	2.88±0.53	t=0.334 P3=0.576	
Anastomosis with internal mammary vessels	0	8(66%)	8(66%)	χ^2^=0.444 P3=0.505	
Anastomosis with the thoracodorsal vessels	0	4(34%)	4(34%)		

Isolated mastectomy flap necrosis, limited to the flap edges, occurred in 2 patients (16.7%) from Group A. In this study, mesh reinforcement was systematically applied to all MS-TRAM flap donor sites (Group A, 100%) to restore the integrity of the rectus sheath. DIEP flaps (Groups B and C) were managed without routine mesh reinforcement. However, specific cases in the DIEP groups presented with pre-existing abdominal wall compromise, including diastasis and weakness, which warranted mesh support. This included 4 of 12 patients (34%) in the conventional DIEP group (Group B) and 2 of 12 patients (16.7%) in the delayed DIEP group (Group C). Statistical analysis demonstrated a significant difference in mesh use between Group A and Group B (p = 0.015) and Group A and Group C (p = 0.06), suggesting that the choice of flap procedure influences the need for mesh support **(**Table 3**).** In our study, only one case with a delayed free DIEP flap underwent a contralateral breast reduction.

**Table 3:** Complication rates between the groups

	Group A N=12	Group B N=12	Group C N=12	Within-group significance
Total flap loss	2(16.7%)	2(16.7%)	0	P1=1.0 P2=1.0 P3=1.0
Partial flap loss	2(16.7%)	2(16.7%)	0	P1=1.0 P2=1.0 P3=1.0
Return to operative time after 48 h	0	2(16.7%)	0	P1=1.0 P2=1.0 P3=1.0
Fat necrosis	2(16.7%)	2(16.7%)	0	P1=1.0 P2=1.0 P3=1.0
Venous congestion	4(34%)	4(34%)	0	P1=1.0 P2=1.0 P3=1.0
Abdominal comorbidity	2(16.7%)	0	0	P1=1.0 P2=1.0 P3=1.0
Redo-venous anastomosis	0	4(34%)	0	P1=1.0 P2=1.0 P3=1.0
Venous supercharge with SIEV	0	4(34%)	0	P1=1.0 P2=1.0 P3=1.0
Mastectomy flap necrosis	2(16.7%)	0	0	P1=1.0 P2=1.0 P3=1.0
Using mesh	12(100%)	4(34%)	2(16.7%)	P1=0.06 P2=0.015* P3=1.0

**Fig. 5:**
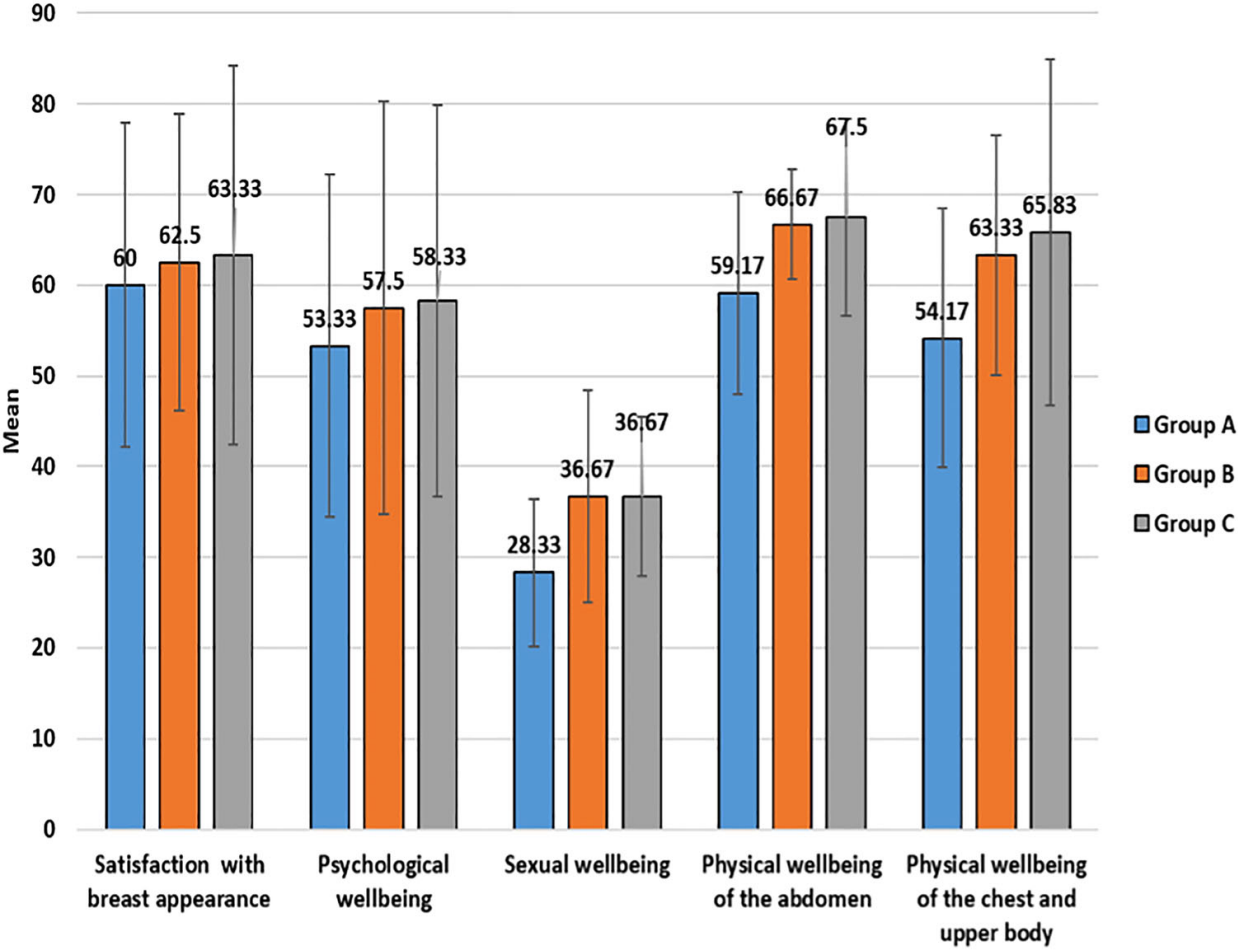
A clustered bar chart across multiple BREAST-Q reconstructive module domains between the three groups

An assessment using the BREAST-Q reconstructive module revealed no statistically significant differences between the three groups in terms of satisfaction with breast appearance, psychological well-being, sexual well-being, physical well-being of the abdomen, and physical well-being of the chest and upper body (*p* > 0.05) (Table 4) (Fig. 5).

**Table 4:** BREAST-Q reconstructive module domains between the three groups

BREAST-Q reconstructive module	Group A N=12	Group B N=12	Group C N=12	Test of significance	Within-group significance
Satisfaction with breast appearance	60.0±17.89	62.50±16.36	63.33±20.89	F=0.053 P=0.949	P1=0.818 P2=0.759 P3=0.939
Psychological well-being	53.33±18.89	57.50±22.75	58.33±21.60	F=0.096 P=0.909	P1=0.738 P2=0.688 P3=0.946
Sexual well-being	28.33±8.17	36.67±11.69	36.67±8.77	F=1.49 P=0.257	P1=0.156 P2=0.156 P3=1.0
Physical well-being of the abdomen	59.17±11.14	66.67±6.06	67.50±10.84	F=1.36 P=0.286	P1=0.197 P2=0.155 P3=0.883
Physical well-being of the chest and upper body	54.17±14.28	63.33±13.29	65.83±19.08	F=0.912 P=0.423	P1=0.330 P2=0.219 P3=0.787

Some clinical cases:

Case 1 (Fig. 6).

**Fig. 6:**
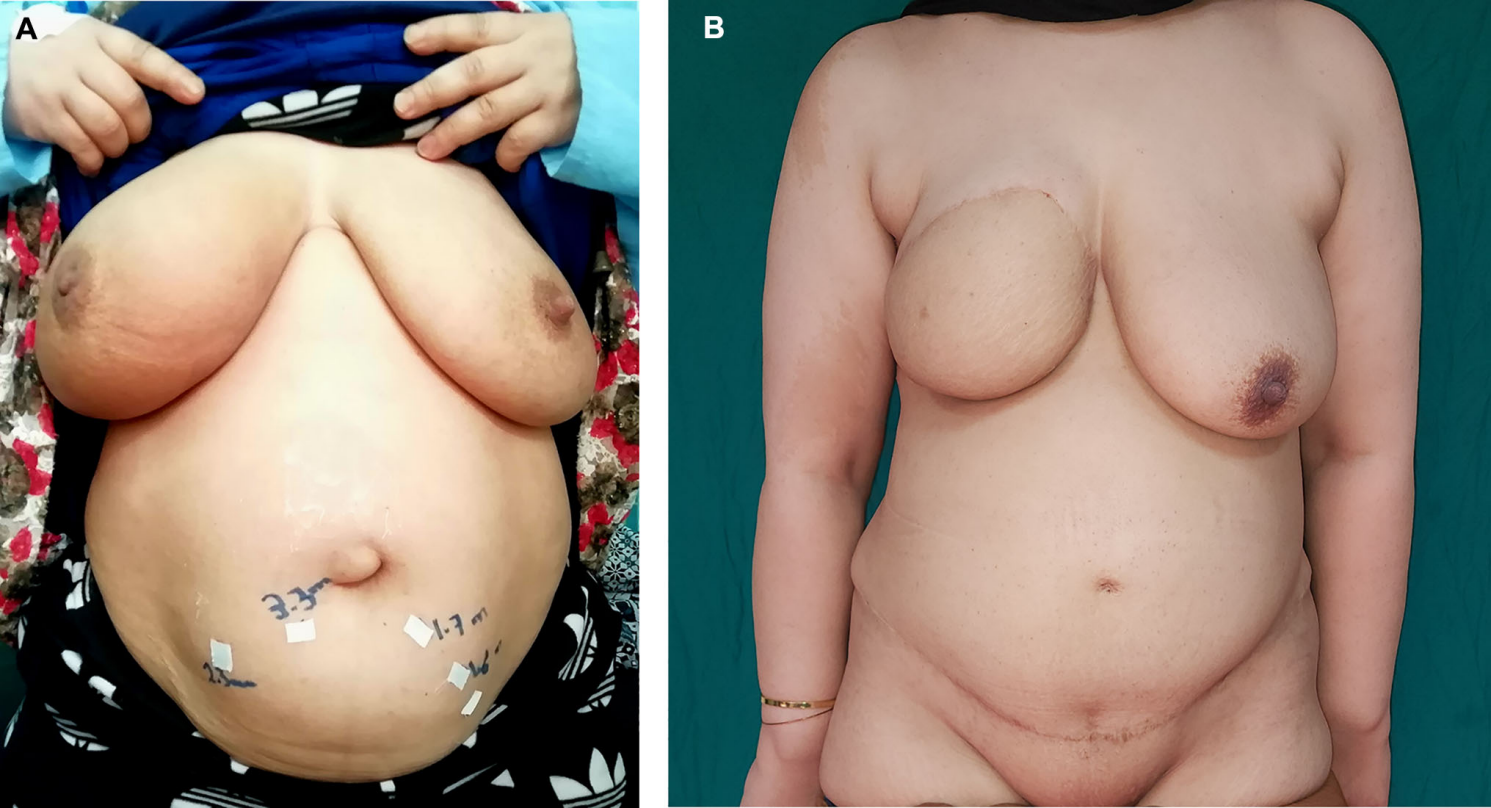
**A** Preoperative view of a 44-year-old female patient with a BMI of 35. She presented with invasive ductal carcinoma of the right breast and underwent neoadjuvant chemotherapy. The surgical procedure included a modified radical mastectomy, axillary clearance, and immediate breast reconstruction utilizing a DIEP free flap (Case 1). **B** Postoperative view, 13 months after free DIEP flap breast reconstruction (Case 1)

Case 2 (Fig. 7).

**Fig. 7:**
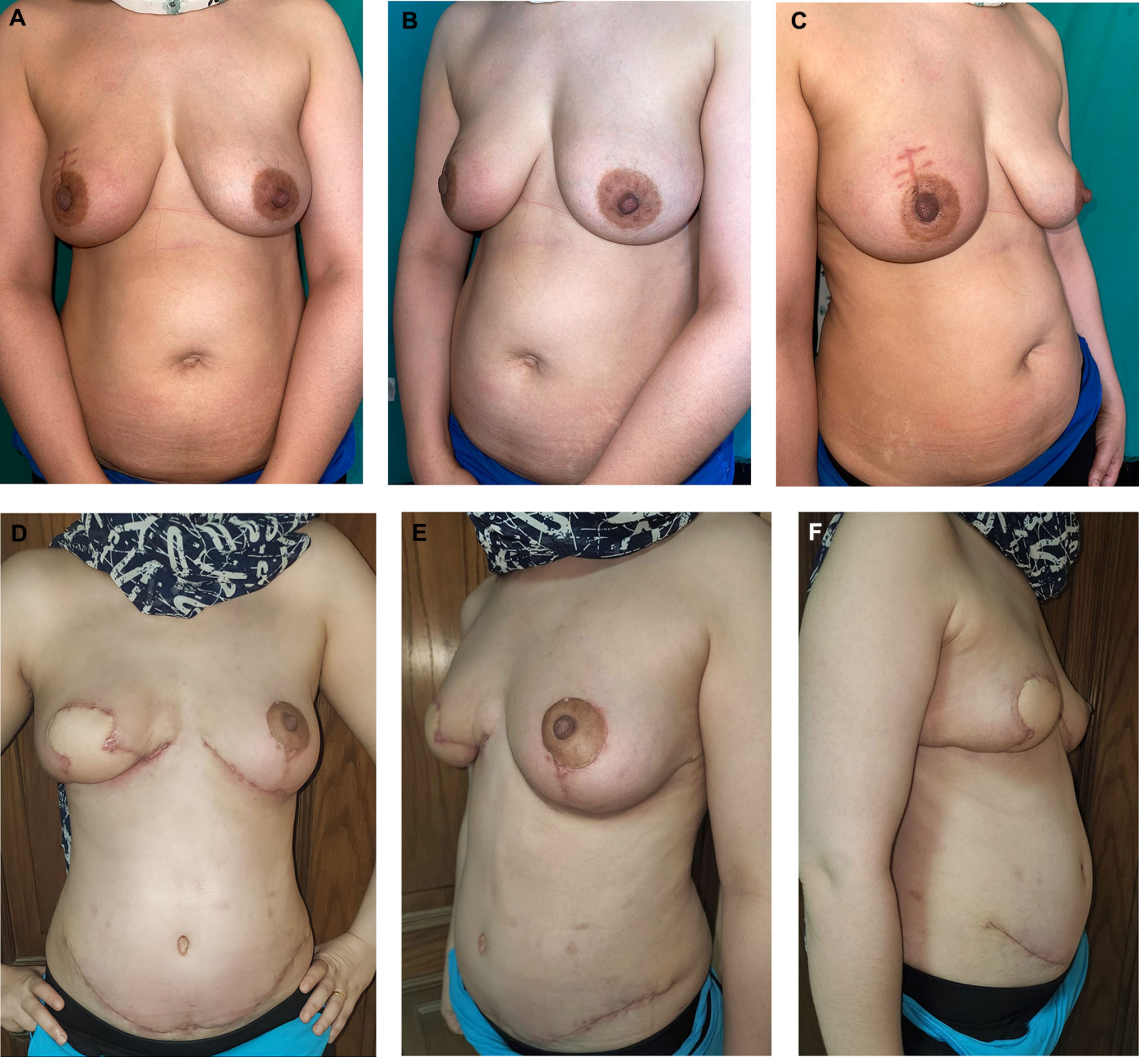
**A** Preoperative anterior view of a 36-year-old female patient with a BMI of 28.9. She presented with invasive ductal carcinoma of the right breast underwent a planned delayed DIEP flap procedure. Initially, the DIEP flap was dissected, preserving the main pedicle. After neoadjuvant chemotherapy, a skin-sparing mastectomy with removal of the nipple–areolar complex with axillary clearance and immediate breast reconstruction using the delayed DIEP free flap along with a contralateral breast reduction. The image shows the anterior view one month postoperatively (Case 2). **B** Preoperative left lateral view (Case 2). **C** Preoperative right lateral view (Case 2). **D** Postoperative anterior view, 9 months after free DIEP flap breast reconstruction (Case 2). **E** Postoperative left lateral view (Case 2). **F** Postoperative right lateral view (Case 2)

## Discussion

Mastectomy rates, especially for women under 50 and over 70, declined until 2005 but have since increased [23]. This reversal, in part due to factors like age, race, and geographic location, coincides with heightened awareness and advancements in breast imaging, leading to improved detection and screening rates for breast cancer [24]. Early-stage breast cancer rates rose from 35.6 to 38.4% between 2000 and 2008 [25]. Many studies approve that autologous tissue reconstruction is the best approach for breast reconstruction. This is because it utilizes native skin and fat, allows for precise shaping and molding, offers long-lasting results, provides a natural appearance and feel, and maximizes the opportunity for sensory restoration [10, 26, 27]. While high-income countries prioritize breast reconstruction after breast cancer surgery due to its benefits for mental health, low- and middle-income countries (LMICs) face challenges [28]. Limited resources and access to specialized equipment often restrict them from using pedicled flap reconstruction techniques, even in community settings. ABR, despite offering superior results and long-term benefits in LMICs, faces challenges due to preference for implants and lack of evidence [29]. The present study provides a comprehensive analysis of QoL outcomes for women who requested immediate, unilateral breast reconstruction using various surgical techniques based on their preference and afterthought counseling during and early post-COVID-19 era. In Egypt, a lower-middle-income country with limited resources, particularly in rural areas, the number of breast reconstruction cases was understandably limited. This was likely due to the ongoing COVID-19 pandemic, which diverted healthcare resources and increased patient anxiety about undergoing elective procedures. Even before and after the COVID-19 pandemic, the number of tertiary centers offering microsurgical breast reconstruction was limited, leading to a greater reliance on pedicled flap techniques. This resulted in improved QoL for patients undergoing immediate reconstruction compared with those who did not [30]. These primary challenges in breast reconstruction align with findings from other studies conducted in similar LMICs [31, 32]. Despite the presence of national cancer registries in many Middle Eastern countries, a notable data gap exists in the area of surgical procedures, as there are no national databases dedicated to tracking them [33]. Patients in Egypt present with advanced-stage breast cancer, posing significant challenges for healthcare providers [34]. Inappropriate therapy is prevalent in many developing countries, driven by factors such as low health budgets, limited access to guidelines, and inadequate healthcare provider training [35]. The selection of the abdominal flap for reconstruction was a multifactorial decision, influenced by surgeon experience, patient preference, and specific patient characteristics. It is important to acknowledge that our center is still in the developing stages of building a microsurgical team, and not all surgeons possess the same level of expertise in these techniques. This influenced the selection of reconstructive procedures, with some surgeons opting for techniques that aligned with their comfort level. The delayed free DIEP flap was particularly valuable for patients with limitations like previous abdominal surgery or higher BMI, allowing for enhanced perfusion without needing supercharging or stacked flaps. This approach ensured that the chosen technique was tailored to both the surgeon’s capabilities and the individual needs of each patient. Our study employed a non-randomized design, mirroring real-world clinical practice where patient preferences and surgeon expertise often guide treatment decisions. Each patient’s reconstructive technique was selected through a collaborative discussion with their surgeon, ensuring personalized treatment plans. While acknowledging the potential for bias in this approach, we believe it offers valuable insights into the factors affecting quality of life outcomes in actual clinical settings. Moreover, the study highlights the constraints posed by limited resources and surgical expertise in Egypt. Access to specialized equipment and the availability of surgeons proficient in microsurgical techniques can influence reconstructive choices. This underscores the need for ongoing investment in surgical training and infrastructure to ensure that all patients have access to the full spectrum of reconstructive options. The author suggests that a lack of cooperation between oncologists and plastic surgeons, coupled with low reimbursement rates for breast reconstruction and limited health insurance coverage, is the primary factors hindering the availability of comprehensive breast surgery data.

Our findings concur with Mijuskovic et al. [36] that duplex ultrasound is highly effective in accurately identifying the optimal dissection side and locating the largest perforators, which is key to optimizing flap harvest and minimizing complications. However, for patients with previous lower abdominal surgeries, CT angiography proved indispensable in evaluating the patency of perforators and superficial vessels. This information is vital for surgical planning and mitigating potential risks associated with compromised blood flow. By strategically combining Duplex US and CTA, we were able to personalize our surgery according to each patient’s unique anatomical and surgical history. This not only allowed us to minimize unnecessary dissection and preserve crucial perforators but also led to a reduction in overall operative time, further improving patient outcomes and recovery. It is valuable in cases with previous abdominal interventions such as liposuction, exploration, or C-section.

It is important to acknowledge that certain factors, such as age, body mass index (BMI), and previous abdominal surgeries, can influence surgical outcomes and potentially affect patient satisfaction. In our study, while age and BMI were comparable across the groups, there was a significant difference in the prevalence of previous abdominal surgeries among the groups. This may have influenced the outcomes of the study, as patients with a history of lower abdominal surgery may be more likely to experience complications. Future studies with larger sample sizes should consider these factors and analyze their potential impact on the outcomes of different surgical techniques.

As highlighted by Rojas et al. [37] and Ochoa et al. [38] age and BMI can significantly influence postoperative outcomes and patient satisfaction. Older patients tend to have lower satisfaction rates, and obese patients may experience delayed wound healing. While our study did not find significant differences in age or BMI between groups, it is crucial to consider these factors in the analysis and interpretation of surgical outcomes [39].

The choice between internal mammary vessels in our study (used in 66% of cases) and thoracodorsal vessels (34%) as recipient vessels was primarily guided by the surgeon’s experience and comfort level with the microvascular anatomy. This approach aligns with recent literature suggesting that the choice of recipient’s vessel does not significantly impact microsurgical outcomes or flap-related complications [40]. Saint-Cyr and colleagues favored the internal mammary perforators for recipient vessels due to their reliability, consistent anatomy, and low complication rates at the recipient site. This approach offers a safe and effective option for surgical procedures [41]. Studies on ischemia time in breast reconstruction have varied. While some studies report shorter times with immediate reconstruction [38, 42], our study aligns with the 60-minute guideline to minimize complications. Our average ischemia times of 42.5 minutes (Group B) and 31 minutes (Group C) fall within this range, contributing to positive outcomes. Our study included diverse groups of breast reconstruction patients, and we observed a wide range of surgical durations, from 5 to 11 h. These variations are consistent with previous research, which highlights the impact of factors such as patient BMI, surgeon experience, and operating room resources on surgical time [38, 43]. Patients with higher BMIs often require more time for surgical dissection, contributing to longer overall procedure times. It is crucial to acknowledge that patient factors, particularly BMI, can significantly influence postoperative outcomes. As highlighted by Ochoa et al. (2012), obese patients face a higher risk of delayed wound healing at both the recipient and donor sites [38]. Microsurgical breast reconstruction, while providing superior outcomes, often necessitates longer hospital stays compared to other reconstruction techniques. Studies have reported average stays ranging from 5 to 8.9 days for DIEP flap procedures, with factors such as immediate versus delayed reconstruction and potential complications influencing the duration [43, 44]. In our study, hospital stays varied from 6 to 12 days, reflecting the complexity of the surgical procedures and the need for extended postoperative monitoring. While some studies report total flap loss rates between 1.3, 2.3, and 4.2% [42, 45, 46], our initial experience demonstrated a higher rate. This discrepancy likely stems from a combination of factors. First, our study, particularly in its early stages, was impacted by the inherent learning curve associated with complex microsurgical techniques. Second, limitations in the availability of advanced intraoperative monitoring techniques, such as ICG angiography, during the initial phase of the study may have hindered early detection and management of potential flap compromise. Third, the absence of a dedicated, fully trained microsurgical team specifically assigned to this study during its initial phases likely played a role. Finally, the relatively low number of cases included in our study may have resulted in a disproportionately larger impact of individual complications on the overall reported rate. This highlights the critical importance of a skilled and collaborative surgical team, along with appropriate support and resources, in achieving consistently successful outcomes in microsurgical breast reconstruction.

The observation of venous congestion in 8 cases across different groups, with no significant intergroup differences, aligns with previous studies, suggesting that venous congestion is a frequent complication in DIEP flaps. A second venous anastomosis, especially one involving the SIEV, has been shown to significantly reduce the risk of this complication [47, 48]. Our results, where redo-venous anastomosis and the SIEV were utilized in 4 cases, further support the potential benefit of this technique. In the delayed Group C, the incidence was nil compared to Group B. Moreover, the minimal occurrence of abdominal comorbidities and the absence of significant differences between groups reinforce the effectiveness of managing venous congestion through additional anastomosis strategies. Partial flap loss was treated conservatively in our study. In DIEP flap breast reconstruction, partial flap loss is influenced by several factors, including prolonged operative time, patient selection, and surgical technique. DIEP flaps demonstrate a greater incidence of partial flap loss when compared to free TRAM flaps [49, 50]. While the use of indocyanine green (ICG) for intraoperative imaging may potentially reduce the risk of partial flap loss [51], its high cost makes it inaccessible to our institution. On the other hand, these rates can be mitigated through careful preoperative planning and the use of intraoperative ICG. Fat necrosis rates in DIEP flap breast reconstruction show significant variability, with incidences reported between 12% and 62.5% [52, 53]. In our study, instances of fat necrosis were minimal in both the DIEP and MS-TRAM groups, and there were no cases observed in the delayed flap group. The authors attribute this to the delay phenomenon, which likely improves vascularity and reduces complications. This enhancement in vascularity and prevention of fat necrosis is consistent with findings from other studies on the delay phenomenon in ABR [54, 55]. Beugels et al. emphasize the efficiency of the delay approach in DIEP flap breast reconstruction, particularly for high-risk cases or those with vascular compromise. The delay procedure in abdominal-based free flap breast reconstruction offers several advantages. It allows for the harvest of a larger volume of well-vascularized tissue, crucial for optimal aesthetic outcomes. By incorporating a preliminary stage, the number of microvascular anastomoses required during the definitive reconstruction is minimized, potentially reducing microvascular complications and overall morbidity. This technique expands the pool of suitable candidates for abdominal-based reconstruction, including patients with previous abdominal surgery, liposuction, or limited perforator vessels who might have been excluded from this reconstructive option traditionally [20]. In contrast, our study explored a modified delay procedure with a 14-day interval between the first and second phases, compared to the 9-day interval used by Beugels et al. [20]. The key advantage of the delayed DIEP flap in our study is to enhance the vascularity and expansion of flap zones. This translates to several significant benefits over the conventional non-delayed DIEP flap. Firstly, it decreases postoperative complications, making it particularly valuable in patients with compromised vasculature due to previous liposuction, abdominal surgeries, or small perforator diameters. Secondly, it allows for harvesting larger flap areas, addressing the needs of patients requiring reconstruction of almost all breast zones. Thirdly, it improves outcomes in patients with high BMI and sizable subcutaneous fat layers, where vascularity can be a concern. While our approach did not utilize ICG mapping, we still achieved successful unilateral breast reconstruction with minimal venous congestion and flap necrosis, further demonstrating the robustness of the delayed technique. This simplified delay method proved to be highly effective, particularly in settings where advanced ICG and perfusion techniques are not available, such as LMICs. By extending the delay period, we observed a reduction in complication rates, thereby enhancing the safety and feasibility of DIEP flap procedures in resource-limited environments. Our findings suggest that the extended delay period can serve as a viable alternative to more technologically demanding methods, ensuring that patients in low-resource settings can still benefit from safe and effective breast reconstruction. This approach aligns with the broader goal of making advanced surgical techniques accessible and reducing disparities in healthcare outcomes. The delayed free DIEP flap was particularly valuable for patients with limitations like previous abdominal surgery or lower BMI, allowing for enhanced perfusion without needing supercharging or stacked flaps. This approach ensured that the chosen technique was tailored to both the surgeon’s capabilities and the individual needs of each patient.

Previous research by Knox and colleagues showed a 3–7% rate of postoperative bulge or hernia after DIEP flap procedures [43]. The use of lateral row perforators was linked to a higher risk of bulging in another study [56]. The authors observed a 16.7% (2/12 patients) rate of small incisional hernias in the MS-TRAM group (Group A) six months postoperatively, despite routine mesh reinforcement of the rectus sheath. This highlights the multifactorial nature of abdominal wall complications. Mesh support utilization varied across groups. While all MS-TRAM patients received prophylactic mesh reinforcement, it was selectively used in 4/12 conventional free DIEP flap patients and 2/12 delayed free DIEP flap patients based on intraoperative assessment of rectus sheath integrity or pre-existing diastasis and abdominal wall weakness. This difference in mesh utilization is a potential confounding factor when comparing outcomes. Our sample size limits a robust analysis of the specific impact of mesh, but the hernias in the MS-TRAM group suggest other factors (surgical technique, patient characteristics, healing) also play a role. Tomita et al. found that microvascular abdominal flap reconstruction produced better results than implant-based methods, except in psychological and sexual well-being [57]. Another study by Cogliandro et al. demonstrated that females undergoing bilateral implant-based reconstruction following nipple-sparing mastectomy reported higher satisfaction levels in terms of appearance, body image, and overall contentment compared to those who had unilateral reconstruction [58]. Many systematic reviews and meta-analyses show that the BREAST-Q questionnaire accurately measures patient satisfaction and QoL after ABR, revealing that autologous reconstruction typically leads to higher satisfaction and well-being than implant-based methods [59, 60]. However, it is important to note that the majority of studies included in these reviews were conducted in high-income countries, and further research is needed to validate these findings in low- and middle-income settings like Egypt. A study conducted across multiple sites used the BREAST-Q mastectomy module to evaluate satisfaction and well-being in women who had undergone mastectomies. The study found that factors such as receiving chemotherapy after surgery and smoking history affected satisfaction levels [61]. Our research indicates that patients generally reported high satisfaction levels concerning breast appearance, psychological well-being, and physical well-being related to the chest and upper body. However, sexual well-being satisfaction was more moderate, likely due to changes in breast sensation and self-esteem throughout the treatment process. Importantly, these results suggest that the specific interventions or treatments received by different patient groups did not lead to significant differences in their overall QoL as assessed by the Breast-Q mastectomy module. Improving patient outcomes, particularly sexual well-being, might involve more than just medical procedures; psychological support and body image counseling could be crucial. While our study highlights the positive impact of immediate breast reconstruction on the QoL of Egyptian women, it also underscores the need for more research on PROMs in LMICs. A review by Malapati et al. revealed a scarcity of studies conducted in LMICs, emphasizing the importance of culturally sensitive and contextually relevant research in these settings [62]. Our study, conducted in Egypt, addresses this gap by providing valuable data on patient-reported outcomes in a resource-constrained environment. These findings contribute to a more comprehensive understanding of the global impact of breast reconstruction and emphasize the importance of considering the unique challenges and contexts of LMICs when evaluating postsurgical outcomes. To our knowledge, this is the first study in Egypt to utilize the BREAST-Q reconstruction module, highlighting the novelty of our approach in evaluating patient-reported outcomes in this context. While our research sheds light on the overall satisfaction levels of patients following breast reconstruction, we acknowledge several limitations. As a single-center non-randomized cohort study with a limited sample size, despite these limitations, this study contributes valuable insights into the impact of immediate unilateral breast reconstruction on QoL in Egyptian women, highlighting the importance of patient-centered care and the need for further research to address the unique challenges of breast reconstruction in resource-constrained settings. The complex challenges hindering the advancement of breast reconstruction in Egypt include fragmented care, resource constraints, and the need for enhanced surgical training. These factors collectively contribute to disparities in healthcare access and limit the availability of advanced reconstructive options. To address these challenges, we advocate for a multidisciplinary approach to breast reconstruction, involving collaboration between surgical oncologists, plastic surgeons, and other healthcare professionals. This collaborative model, while relatively new in the Egyptian healthcare system, holds the potential to significantly improve patient outcomes and satisfaction. The authors are sincerely committed to promoting advanced microsurgical breast reconstruction in Egypt and incorporating the full range of BREAST-Q modules in future studies to gain a more comprehensive understanding of the patient experience and satisfaction following breast reconstruction procedures. This will enable us to better assess the impact of different interventions and contribute to the development of more patient-centered care. Furthermore, the authors believe that addressing the systemic challenges, such as limited access to specialized equipment, the need for enhanced surgical training, and financial constraints faced by patients, is crucial for advancing breast reconstruction in Egypt. By promoting interdisciplinary collaboration, advocating for improved infrastructure, and optimizing resource allocation, we can strive to ensure that breast reconstruction in Egypt reaches its full potential, benefiting both patients and the medical community.

## Conclusion

This Egyptian study examines the immediate unilateral ABR impact on QoL after mastectomy. Despite resource limitations, it found similar QoL outcomes across abdominal flap techniques, suggesting individualization based on patient needs and resources without compromising satisfaction. The study emphasizes personalized approaches, shared decision making, and the BREAST-Q’s effectiveness. While acknowledging limitations, it advocates for larger studies to explore long-term satisfaction factors. Ultimately, this research contributes valuable insights into breast reconstruction in resource-limited settings like Egypt, highlighting tailored surgical approaches and multidisciplinary care.

## References

[1] Ettridge K, Scharling-Gamba K, Miller C, Roder D, Prichard I. Body image and quality of life in women with breast cancer: appreciating the body and its functionality. Body Image. 2022;40:92–102. 34902783 10.1016/j.bodyim.2021.11.001

[2] Brunet J, Price J, Harris C. Body image in women diagnosed with breast cancer: a grounded theory study. Body Image. 2022;41:417–31. 10.1016/j.bodyim.2022.04.012. 35526352 10.1016/j.bodyim.2022.04.012

[3] Ferlay J, Ervik M, Lam F, Laversanne M, Colombet M, Mery L, Piñeros M, Znaor A, Soerjomataram I, Bray F. Global cancer observatory: cancer today. International Agency for Research on Cancer. 2024. Available from: https://gco.iarc.who.int/today, Accessed 14 Feb 2025.

[4] Abdelrahman SM, Ibraheem MH, Allam H, Sewram V. Factors affecting the quality of life in women post mastectomy for breast cancer in Baheya foundation (Egypt): a retrospective cohort study. BMC Womens Health. 2025;25(1):43. 10.1186/s12905-025-03571-z. 39893412 10.1186/s12905-025-03571-z PMC11786333

[5] Kummerow KL, Du L, Penson DF, Shyr Y, Hooks MA. Nationwide trends in mastectomy for early-stage breast cancer. JAMA Surg. 2015;150(1):9–16. 10.1001/jamasurg.2014.2895. 25408966 10.1001/jamasurg.2014.2895

[6] Zehra S, Doyle F, Barry M, Walsh S, Kell MR. Health-related quality of life following breast reconstruction compared to total mastectomy and breast-conserving surgery among breast cancer survivors: a systematic review and meta-analysis. Breast Cancer. 2020;27(4):534–66. 10.1007/s12282-020-01076-1. 32162181 10.1007/s12282-020-01076-1

[7] Eltahir Y, Werners LLCH, Dreise MM, Zeijlmans van Emmichoven IA, Werker PMN, de Bock GH. Which breast is the best? Successful autologous or alloplastic breast reconstruction: patient-reported quality-of-life outcomes. Plast Reconstr Surg. 2015;135(1):43–50. 10.1097/PRS.0000000000000804. 25539295 10.1097/PRS.0000000000000804

[8] Munhoz AM, Montag E, Filassi JR, Gemperli R. Current approaches to managing partial breast defects: the role of conservative breast surgery reconstruction. Anticancer Res. 2014;34(3):1099–114. 24596348

[9] Schop SJ, Joosen MEM, Wolswijk T, Heuts EM, van der Hulst RRWJ, Piatkowski de Grzymala AA. Quality of life after autologous fat transfer additional to prosthetic breast reconstruction in women after breast surgery: a systematic review. Eur J Surg Oncol. 2021;47(4):772–7. 10.1016/j.ejso.2020.10.021. 33243607 10.1016/j.ejso.2020.10.021

[10] Santosa KB, Qi J, Kim HM, Hamill JB, Wilkins EG, Pusic AL. Long-term patient-reported outcomes in postmastectomy breast reconstruction. JAMA Surg. 2018;153(10):891–9. 10.1001/jamasurg.2018.1677. 29926096 10.1001/jamasurg.2018.1677 PMC6233781

[11] Coriddi M, Shenaq D, Kenworthy E, et al. Autologous breast reconstruction after failed implant-based reconstruction: evaluation of surgical and patient-reported outcomes and quality of life. Plast Reconstr Surg. 2019;143(2):373–9. 10.1097/PRS.0000000000005197. 30688876 10.1097/PRS.0000000000005197 PMC6352728

[12] Tachi M, Yamada A. Choice of flaps for breast reconstruction. Int J Clin Oncol. 2005;10(5):289–97. 10.1007/s10147-005-0527-4. 16247654 10.1007/s10147-005-0527-4

[13] Hartrampf CR, Scheflan M, Black PW. Breast reconstruction with a transverse abdominal island flap. Plast Reconstr Surg. 1982;69(2):216–25. 10.1097/00006534-198202000-00006. 6459602 10.1097/00006534-198202000-00006

[14] Allen RJ, Treece P. Deep inferior epigastric perforator flap for breast reconstruction. Ann Plast Surg. 1994;32(1):32–8. 10.1097/00000637-199401000-00007. 8141534 10.1097/00000637-199401000-00007

[15] Wang XL, Liu LB, Song FM, Wang QY. Meta-analysis of the safety and factors contributing to complications of MS-TRAM, DIEP, and SIEA flaps for breast reconstruction. Aesthet Plast Surg. 2014;38(4):681–91. 10.1007/s00266-014-0333-3. 10.1007/s00266-014-0333-3 24902911

[16] Greenspun DT, Koolen PGL, Lee BT, Lin SJ, Erhard HA. The stacked hemiabdominal extended perforator flap for autologous breast reconstruction. Plast Reconstr Surg. 2019;144(5):923e–4e. 10.1097/PRS.0000000000006103. 31397789 10.1097/PRS.0000000000006103

[17] Patel NG, Rozen WM, Chow WT, et al. Stacked and bipedicled abdominal free flaps for breast reconstruction: considerations for shaping. Gland Surg. 2016;5(2):115–21. 10.3978/j.issn.2227-684X.2016.02.03. 27047780 10.3978/j.issn.2227-684X.2016.02.03 PMC4791347

[18] Mayo JL, Allen RJ, Sadeghi A. Four-flap breast reconstruction: bilateral stacked DIEP and PAP flaps. Plast Reconstr Surg Glob Open. 2015;3(5): e383. 10.1097/GOX.0000000000000353. 26090273 10.1097/GOX.0000000000000353 PMC4457246

[19] Haddock NT, Kelling JA, Teotia SS. Simultaneous circumferential body lift and four-flap breast reconstruction using deep inferior epigastric perforator and lumbar artery perforator flaps. Plast Reconstr Surg. 2021;147(6):936e–9e. 10.1097/PRS.0000000000007992. 34019499 10.1097/PRS.0000000000007992

[20] Beugels J, Levine JL, Vasile JV, Craigie JE, Allen RJ. The delay procedure in deep inferior epigastric artery perforator flap breast reconstruction. Plast Reconstr Surg. 2024;153(6):1063e–72e. 10.1097/PRS.0000000000010837. 37335555 10.1097/PRS.0000000000010837

[21] Pusic AL, Klassen AF, Scott AM, Klok JA, Cordeiro PG, Cano SJ. Development of a new patient-reported outcome measure for breast surgery: the BREAST-Q. Plast Reconstr Surg. 2009;124(2):345–53. 10.1097/PRS.0b013e3181aee807. 19644246 10.1097/PRS.0b013e3181aee807

[22] Fong A, Park HS, Ross DA, Rozen WM. Preoperative planning of unilateral breast reconstruction with pedicled transverse rectus abdominis myocutaneous (TRAM) flaps: a pilot study of perforator mapping. Gland Surg. 2023;12(3):366–73. 10.21037/gs-22-529. 37057040 10.21037/gs-22-529 PMC10086775

[23] Dragun AE, Huang B, Tucker TC, Spanos WJ. Increasing mastectomy rates among all age groups for early stage breast cancer: a 10-year study of surgical choice. Breast J. 2012;18(4):318–25. 10.1111/j.1524-4741.2012.01245.x. 22607016 10.1111/j.1524-4741.2012.01245.x

[24] Akram M, Iqbal M, Daniyal M, Khan AU. Awareness and current knowledge of breast cancer. Biol Res. 2017;50(1):33. 10.1186/s40659-017-0140-9. 28969709 10.1186/s40659-017-0140-9 PMC5625777

[25] Mahmood U, Hanlon AL, Koshy M, et al. Increasing national mastectomy rates for the treatment of early stage breast cancer. Ann Surg Oncol. 2013;20(5):1436–43. 10.1245/s10434-012-2732-5. 23135312 10.1245/s10434-012-2732-5

[26] Nahabedian MY, Patel K. Autologous flap breast reconstruction: Surgical algorithm and patient selection. J Surg Oncol. 2016;113(8):865–74. 10.1002/jso.24208. 26918920 10.1002/jso.24208

[27] Garza R, Ochoa O, Chrysopoulo M. Post-mastectomy breast reconstruction with autologous tissue: current methods and techniques. Plast Reconstr Surg Glob Open. 2021;9(2): e3433. 10.1097/GOX.0000000000003433. 33680677 10.1097/GOX.0000000000003433 PMC7929567

[28] Albornoz CR, Bach PB, Mehrara BJ, et al. A paradigm shift in U.S. Breast reconstruction: increasing implant rates. Plast Reconstr Surg. 2013;131(1):15–23. 10.1097/PRS.0b013e3182729cde. 23271515 10.1097/PRS.0b013e3182729cde

[29] Shah V, Soh CL, Chhatwal K, et al. Autologous breast reconstruction in low- and middle-income countries (LMICs): a systematic review of current practices and challenges. Minerva Surg. 2024;79(1):73–81. 10.23736/S2724-5691.23.10111-0. 38381032 10.23736/S2724-5691.23.10111-0

[30] Denewer A, Farouk O, Kotb S, Setit A, Abd El-Khalek S, Shetiwy M. Quality of life among Egyptian women with breast cancer after sparing mastectomy and immediate autologous breast reconstruction: a comparative study. Breast Cancer Res Treat. 2012;133(2):537–44. 10.1007/s10549-011-1792-8. 21956212 10.1007/s10549-011-1792-8

[31] Sinaei F, Zendehdel K, Adili M, Ardestani A, Montazeri A, Mohagheghi MA. Association between breast reconstruction surgery and quality of life in Iranian breast cancer patients. Acta Med Iran. 2017;55(1):35–41. 28188941

[32] Forouzanfar MH, Foreman KJ, Delossantos AM, et al. Breast and cervical cancer in 187 countries between 1980 and 2010: a systematic analysis. Lancet. 2011;378(9801):1461–84. 10.1016/S0140-6736(11)61351-2. 21924486 10.1016/S0140-6736(11)61351-2

[33] Khan N, Saleem HY, Huayllani MT, Boczar D, Forte AJ. Breast reconstruction in the middle east: a controversial topic. Plast Reconstr Surg. 2020;145(5):1011e–2e. 10.1097/PRS.0000000000006758. 32332576 10.1097/PRS.0000000000006758

[34] El Saghir NS. Responding to the challenges of breast cancer in egypt and other arab countries. J Egypt Natl Canc Inst. 2008;20(4):309–12. 20571588

[35] Salem AA, Salem MA, Abbass H. Breast cancer: surgery at the South Egypt cancer institute. Cancers. 2010;2(4):1771–8. 10.3390/cancers2041771. 24281200 10.3390/cancers2031771 PMC3840361

[36] Mijuskovic B, Tremp M, Heimer MM, et al. Color Doppler ultrasound and computed tomographic angiography for perforator mapping in DIEP flap breast reconstruction revisited: a cohort study. J Plast Reconstr Aesthet Surg. 2019;72(10):1632–9. 10.1016/j.bjps.2019.06.008. 31375431 10.1016/j.bjps.2019.06.008

[37] Rojas KE, Matthews N, Raker C, et al. Body mass index (BMI), postoperative appearance satisfaction, and sexual function in breast cancer survivorship. J Cancer Surviv. 2018;12(1):127–33. 10.1007/s11764-017-0651-y. 29043480 10.1007/s11764-017-0651-y

[38] Ochoa O, Chrysopoulo M, Nastala C, Ledoux P, Pisano S. Abdominal wall stability and flap complications after deep inferior epigastric perforator flap breast reconstruction: does body mass index make a difference? Analysis of 418 patients and 639 flaps. Plast Reconstr Surg. 2012;130(1):21e–33e. 10.1097/PRS.0b013e3182547d09. 22743936 10.1097/PRS.0b013e3182547d09

[39] Jiang L, Ji X, Liu W, Qi C, Zhai X. BREAST-Q-based survey of the satisfaction and health status of patients with breast reconstruction. Aesthet Plast Surg. 2023;47(6):2295–303. 10.1007/s00266-023-03642-2. 10.1007/s00266-023-03642-2 PMC10784367 37697090

[40] Lemdani MS, Crystal DT, Ewing JN, et al. reevaluation of recipient vessel selection in breast free flap reconstruction. Microsurgery. 2024;44(7): e31222. 10.1002/micr.31222. 39340204 10.1002/micr.31222

[41] Saint-Cyr M, Chang DW, Robb GL, Chevray PM. Internal mammary perforator recipient vessels for breast reconstruction using free TRAM, DIEP, and SIEA flaps. Plast Reconstr Surg. 2007;120(7):1769–73. 10.1097/01.prs.0000287132.35433.d6. 18090738 10.1097/01.prs.0000287132.35433.d6

[42] Prantl L, Moellhoff N, von Fritschen U, et al. Immediate versus secondary DIEP flap breast reconstruction: a multicenter outcome study. Arch Gynecol Obstet. 2020;302(6):1451–9. 10.1007/s00404-020-05779-w. 32895743 10.1007/s00404-020-05779-w PMC7584555

[43] Knox ADC, Ho AL, Leung L, et al. Comparison of outcomes following autologous breast reconstruction using the DIEP and Pedicled TRAM flaps: a 12-year clinical retrospective study and literature review. Plast Reconstr Surg. 2016;138(1):16–28. 10.1097/PRS.0000000000001747. 26267400 10.1097/PRS.0000000000001747

[44] Chevray PM. Breast reconstruction with superficial inferior epigastric artery flaps: a prospective comparison with TRAM and DIEP flaps. Plast Reconstr Surg. 2004;114(5):1077–83. 10.1097/01.prs.0000135328.88101.53. 15457015 10.1097/01.prs.0000135328.88101.53

[45] Bajaj AK, Chevray PM, Chang DW. Comparison of donor-site complications and functional outcomes in free muscle-sparing TRAM flap and free DIEP flap breast reconstruction. Plast Reconstr Surg. 2006;117(3):737–46. 10.1097/01.prs.0000200062.97265.fb. 16525258 10.1097/01.prs.0000200062.97265.fb

[46] Cho MJ, Haddock NT, Teotia SS. Clinical decision making using CTA in conjoined, bipedicled DIEP and SIEA for unilateral breast reconstruction. J Reconstr Microsurg. 2020;36(4):241–6. 10.1055/s-0039-3400542. 31801159 10.1055/s-0039-3400542

[47] Pignatti M, Pinto V, Giorgini FA, et al. Meta-analysis of the effects of venous super-drainage in deep inferior epigastric artery perforator flaps for breast reconstruction. Microsurgery. 2021;41(2):186–95. 10.1002/micr.30682. 33170970 10.1002/micr.30682

[48] Tran NV, Buchel EW, Convery PA. Microvascular complications of DIEP flaps. Plast Reconstr Surg. 2007;119(5):1397–405. 10.1097/01.prs.0000256045.71765.96. 17415232 10.1097/01.prs.0000256045.71765.96

[49] Lie KH, Barker AS, Ashton MW. A classification system for partial and complete DIEP flap necrosis based on a review of 17,096 DIEP flaps in 693 articles including analysis of 152 total flap failures. Plast Reconstr Surg. 2013;132(6):1401–8. 10.1097/01.prs.0000434402.06564.bd. 24281570 10.1097/01.prs.0000434402.06564.bd

[50] Busic V, Das-Gupta R, Mesic H, Begic A. The deep inferior epigastric perforator flap for breast reconstruction, the learning curve explored. J Plast Reconstr Aesthet Surg. 2006;59(6):580–4. 10.1016/j.bjps.2005.04.061. 16817256 10.1016/j.bjps.2005.04.061

[51] Casey WJ, Connolly KA, Nanda A, Rebecca AM, Perdikis G, Smith AA. Indocyanine green laser angiography improves deep inferior epigastric perforator flap outcomes following abdominal suction lipectomy. Plast Reconstr Surg. 2015;135(3):491e–7e. 10.1097/PRS.0000000000000964. 25719713 10.1097/PRS.0000000000000964

[52] Bhullar H, Hunter-Smith DJ, Rozen WM. Fat necrosis after DIEP flap breast reconstruction: a review of perfusion-related causes. Aesthet Plast Surg. 2020;44(5):1454–61. 10.1007/s00266-020-01784-1. 10.1007/s00266-020-01784-1 32445045

[53] Malagón-López P, Vilà J, Carrasco-López C, et al. Intraoperative indocyanine green angiography for fat necrosis reduction in the deep inferior epigastric perforator (DIEP) flap. Aesthet Surg J. 2019;39(4):NP45–54. 10.1093/asj/sjy256. 30358820 10.1093/asj/sjy256

[54] Christiano JG, Rosson GD. Clinical experience with the delay phenomenon in autologous breast reconstruction with the deep inferior epigastric artery perforator flap. Microsurgery. 2010;30(7):526–31. 10.1002/micr.20787. 20853336 10.1002/micr.20787

[55] Melancon DM, Yoo D, Stern-Buchbinder Z, Morin S, St Hilaire H, Allen RJ. A unique advantage of a 24-hour surgical delay in autologous breast reconstruction. Plast Reconstr Surg Glob Open. 2024;12(10): e6231. 10.1097/GOX.0000000000006231. 39386096 10.1097/GOX.0000000000006231 PMC11463200

[56] Grünherz L, Wolter A, Andree C, Thamm O. Invited response on: breast reconstruction with SIEA flaps: an alternative in selected cases. Aesthet Plast Surg. 2020;44(2):621–2. 10.1007/s00266-020-01640-2. 10.1007/s00266-020-01640-2 32043164

[57] Tomita S, Nagai K, Matsunaga N, Kerckhove M, Fujii M, Terao Y. Detailed analysis of three major breast reconstructions using BREAST-Q responses from 1001 patients. Aesthet Surg J. 2023;43(11):NP888–97. 10.1093/asj/sjad205. 37392431 10.1093/asj/sjad205

[58] Cogliandro A, Salzillo R, Barone M, Tenna S, Cagli B, Persichetti P. Direct-to-implant breast reconstruction after unilateral and bilateral mastectomy: cross-sectional study of patient satisfaction and quality of life with BREAST-Q. Aesthet Plast Surg. 2023;47(1):43–9. 10.1007/s00266-022-02986-5. 10.1007/s00266-022-02986-5 35927501

[59] Toyserkani NM, Jørgensen MG, Tabatabaeifar S, Damsgaard T, Sørensen JA. Autologous versus implant-based breast reconstruction: a systematic review and meta-analysis of Breast-Q patient-reported outcomes. J Plast Reconstr Aesthet Surg. 2020;73(2):278–85. 10.1016/j.bjps.2019.09.040. 31711862 10.1016/j.bjps.2019.09.040

[60] Seth I, Seth N, Bulloch G, Rozen WM, Hunter-Smith DJ. Systematic review of breast-Q: a tool to evaluate post-mastectomy breast reconstruction. Breast Cancer. 2021;13:711–24. 10.2147/BCTT.S256393. 34938118 10.2147/BCTT.S256393 PMC8687446

[61] Alghamdi H, Alhefdhi A, Fayi KA, et al. The satisfaction and quality of life of patients after breast reconstruction: a cross-sectional multicenter study comparing immediate, delayed, and nonreconstructive outcomes. Ann Plast Surg. 2024;93(4):425–9. 10.1097/SAP.0000000000004040. 38984731 10.1097/SAP.0000000000004040

[62] Malapati SH, Hyland CJ, Liang G, et al. Use of patient-reported outcome measures after breast reconstruction in low-and middle-income countries: a scoping review. J Patient Rep Outcomes. 2024;8(1):25. 10.1186/s41687-024-00687-y. 38416222 10.1186/s41687-024-00687-y PMC10899941

